# Supplementary material for: The parafascicular thalamus steers attention to facilitate learning
Source: bioRxiv. 2026 Jun 19:2026.06.18.733204. Preprint. [Version 1] doi: 10.64898/2026.06.18.733204 (PMC13308099; doi:10.64898/2026.06.18.733204)
Supplement: 1 [file NIHPP2026.06.18.733204v1-supplement-1.pdf]

bioRxiv preprint doi: https://doi.org/10.64898/2026.06.18.733204; this version posted June 19, 2026. The copyright holder for this preprint (which was not certified by peer review) is the author/funder, who has granted bioRxiv a license to display the preprint in perpetuity. It is made available under a CC-BY 4.0 International license.

Supplemental Material

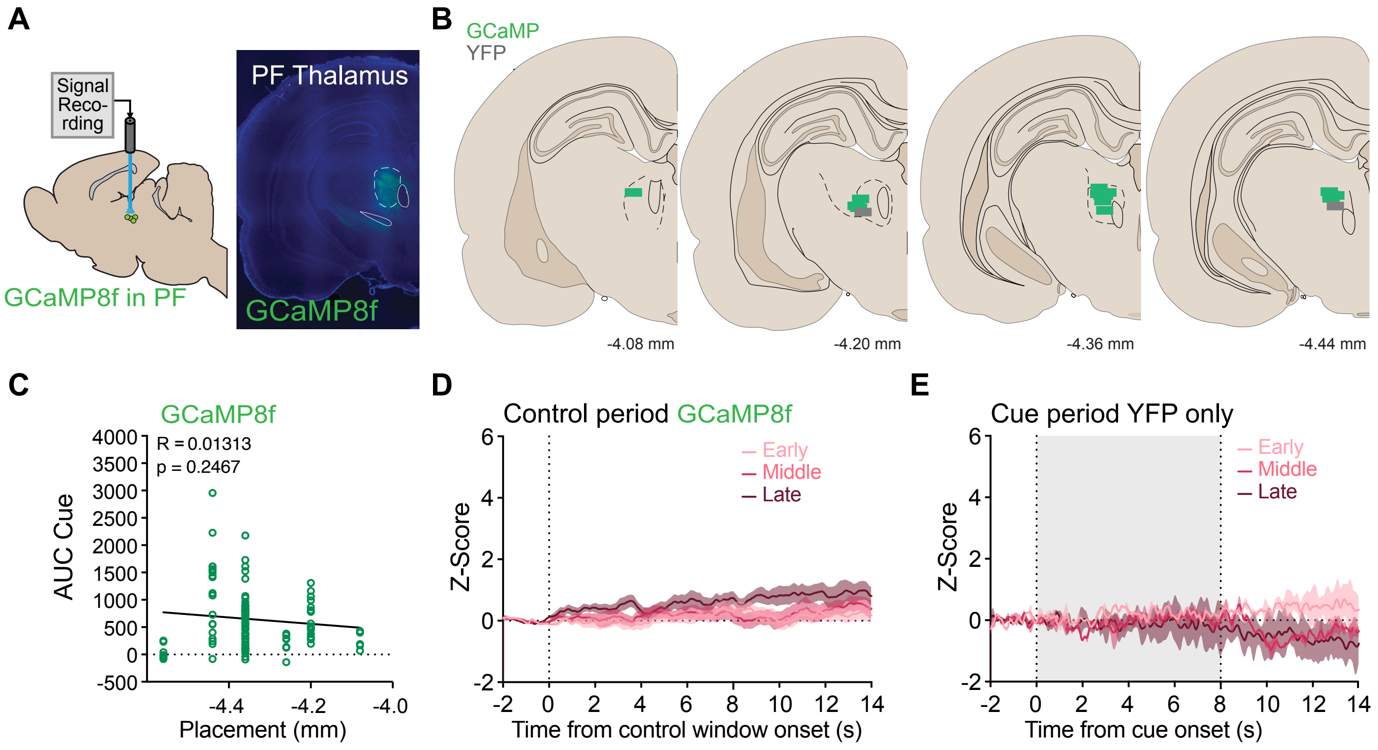

Fig S1 | Fiber photometry recording histology and control signals

A) Representative image of GCaMP expression and fiber placement. B) Fiber placements in the parafasicicular (PF) thalamus. C) We found no correlation between recording location along the anterior-posterior axis and the magnitude of GCaMP cue responses (R=0.01313, p=0.2467). D) Recordings of PF GCaMP during control windows (within the inter-trial interval) showed no changes. E) Control photometry recordings made from a YFP-only virus showed no change in signal during cue presentations. Data shown reflect mean +/- SEM.

bioRxiv preprint doi: https://doi.org/10.64898/2026.06.18.733204; this version posted June 19, 2026. The copyright holder for this preprint (which was not certified by peer review) is the author/funder, who has granted bioRxiv a license to display the preprint in perpetuity. It is made available under a CC-BY 4.0 International license.

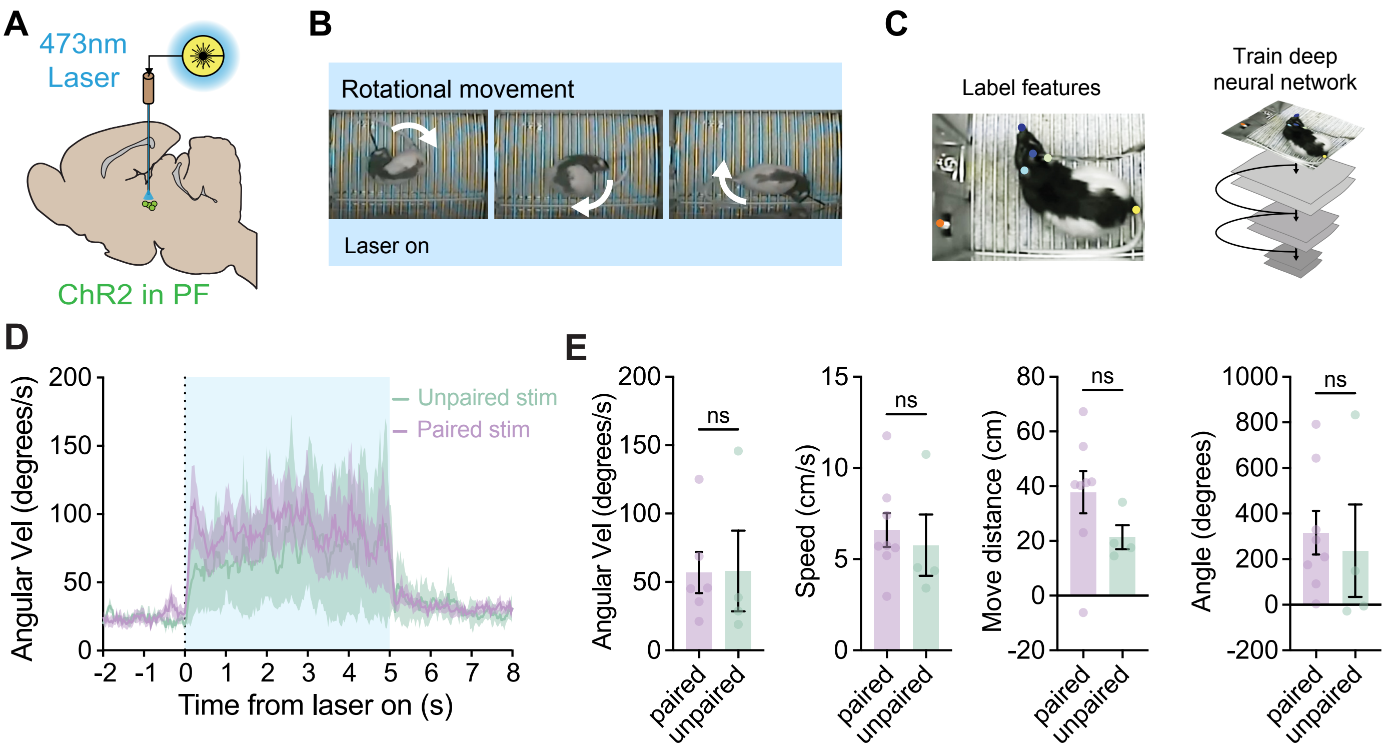

Fig S2 | Turning behavior during unpaired PF activation

A) ChR2-YFP was expressed and optic fibers were implanted unilaterally in the PF thalamus. Optogenetic stimulation (20 Hz, 5 sec) was delivered either during the cue period (paired, n=8) or inter-trial-interval (unpaired, n=4). B) Representative images of rotational movement while the laser was on. Rats curled and turned their entire body in circles, directed by their head. C) Schematic of behavioral analysis via DeepLabCut. D) Average angular velocity on the last day for paired and unpaired stimulation conditions. E) There were no significant differences in average angular velocity, average speed, move distance, or cumulative angle between paired stimulation and unpaired stimulation groups. Data shown reflect mean +/- SEM.

bioRxiv preprint doi: https://doi.org/10.64898/2026.06.18.733204; this version posted June 19, 2026. The copyright holder for this preprint (which was not certified by peer review) is the author/funder, who has granted bioRxiv a license to display the preprint in perpetuity. It is made available under a CC-BY 4.0 International license.

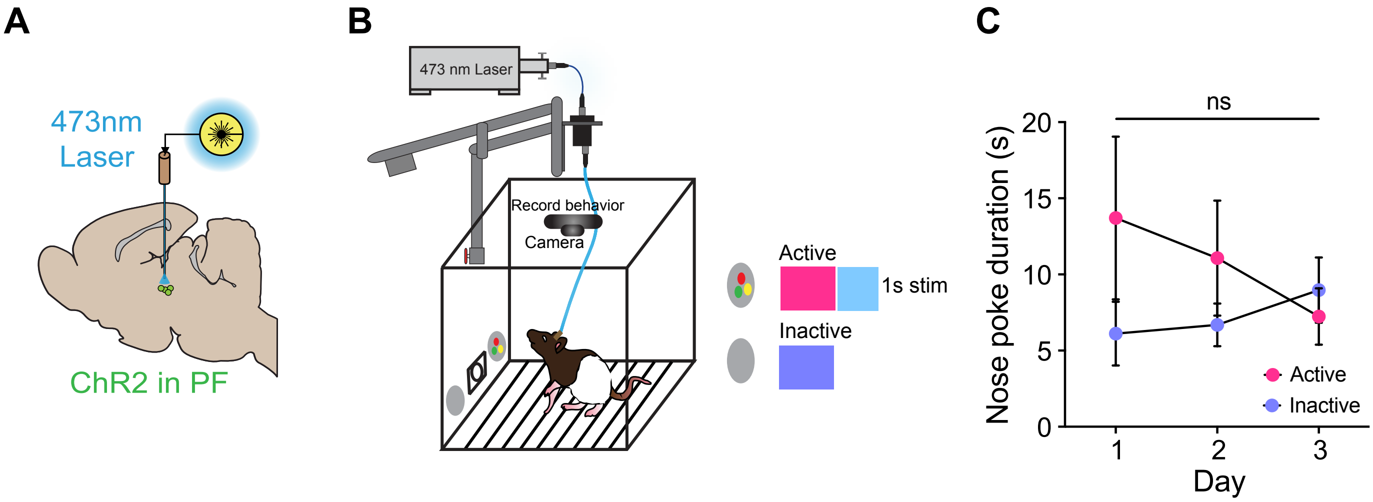

Fig S3 | PF stimulation is not reinforcing

A) ChR2 was expressed unilaterally in the PF thalamus. B) Rats (n=11) were given the opportunity to self-stimulate the PF, where active nose pokes coincided with a brief visual cue inside the poke and a 1-s 20 Hz laser pulse. C) There was no difference in nose poke duration between active and inactive nose pokes. Data shown reflect mean +/- SEM.
